# Supplementary material for: Loss of a proteostatic checkpoint in intestinal stem cells contributes to age-related epithelial dysfunction
Source: Nat Commun. 2019 Mar 5;10:1050. doi: 10.1038/s41467-019-08982-9 (PMC6401111; doi:10.1038/s41467-019-08982-9)
Supplement: Supplementary file 1 — Supplementary Information [file 41467_2019_8982_MOESM1_ESM.pdf]

Supplementary Information for

Loss of a proteostatic checkpoint in intestinal stem cells contributes to age-related epithelial dysfunction

Imilce A. Rodriguez-Fernandez[1,2], Yanyan Qi[1] and Heinrich Jasper[1,2,3*]

[1]Buck Institute for Research on Aging, 8001 Redwood Boulevard, Novato, CA 94945-1400, USA.
[2]Immunology Discovery, Genentech, Inc., 1 DNA Way, South San Francisco, California 94080, USA.
[3]Leibniz Institute on Aging - Fritz Lipmann Institute, Jena, Germany, 07745.
[*]correspondence and requests for materials should be addresses to H.J. (email: jasperh@gene.com)

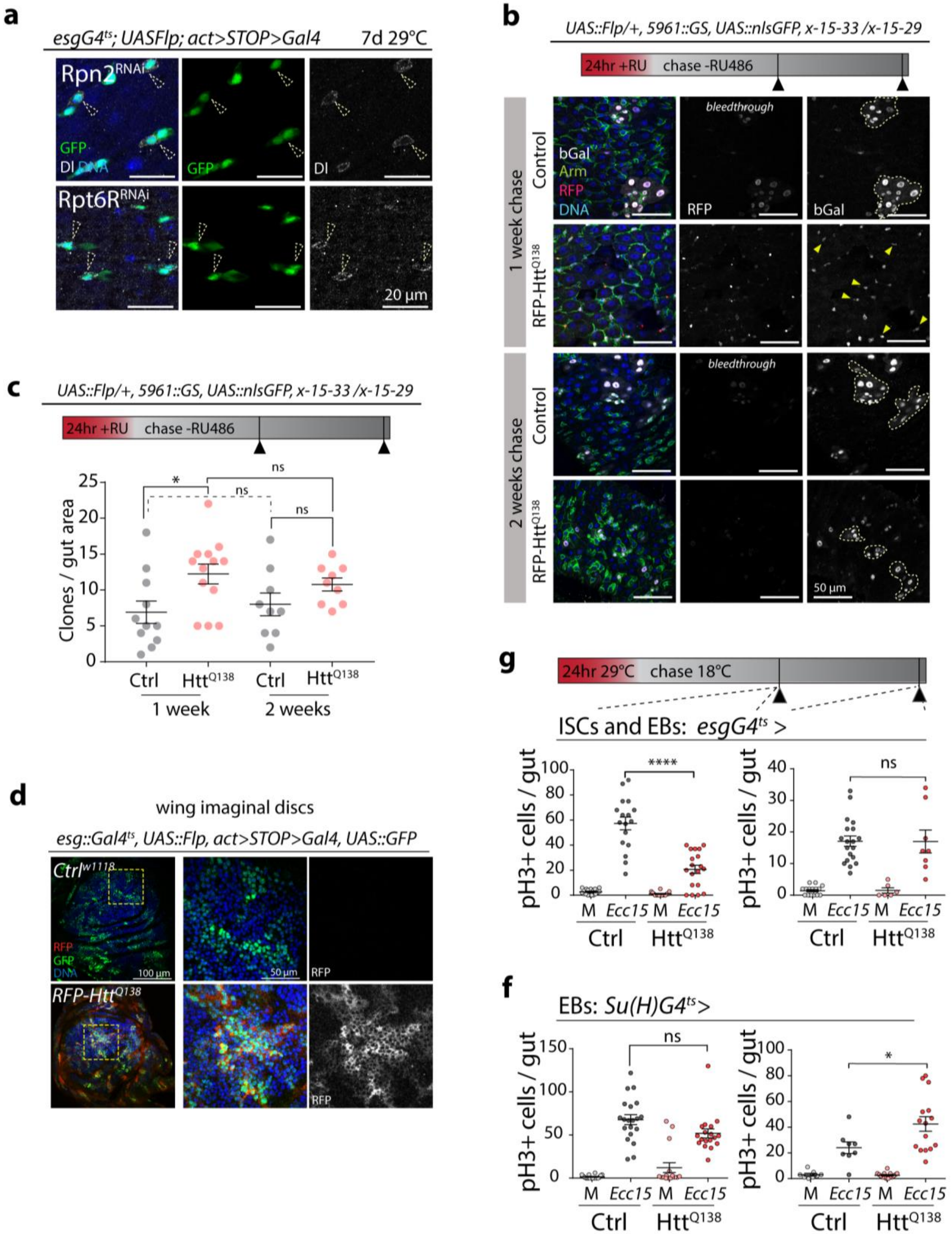
a
esgG4ts; UASFlp; act>STOP>Gal4
7d 29°C
Rpn2RNAi
GFP
Dl DNA
GFP
Dl
Rpt6RRNAi
20 µm
b
UAS::Flp/+, 5961::GS, UAS::nlsGFP, x-15-33 /x-15-29
24hr +RU
chase -RU486
1 week chase
Control
bGal
Arm
RFP
DNA
bleedthrough
RFP
bGal
RFP-HttQ138
2 weeks chase
Control
bleedthrough
RFP-HttQ138
50 µm
c
UAS::Flp/+, 5961::GS, UAS::nlsGFP, x-15-33 /x-15-29
24hr +RU
chase -RU486
Clones / gut area
ns
ns
ns
Ctrl
HttQ138
Ctrl
HttQ138
1 week
2 weeks
d
wing imaginal discs
esg::Gal4ts, UAS::Flp, act>STOP>Gal4, UAS::GFP
Ctrlw1118
RFP
GFP
DNA
100 µm
50 µm
RFP
RFP-HttQ138
RFP
g
24hr 29°C
chase 18°C
ISCs and EBs: esgG4ts >
pH3+ cells / gut
****
ns
M
Ecc15
Ctrl
HttQ138
f
EBs: Su(H)G4ts>
pH3+ cells / gut
ns
M
Ecc15
Ctrl
HttQ138

Supplementary Figure 1. a, Representative area of 6-day-old fly posterior midguts carrying GFP-marked lineages (green) derived from ISCs expressing *Rpn2*^{RNAi} or *Rpt6R*^{RNAi} (using a Flp-out strategy). Images of control lineages and quantification of cell numbers per clones are found in Fig. 1A. Immunohistochemistry was performed with anti-Delta antibody to label ISCs (white). **b**, Lower magnification of the lineages shown in Fig. 1d. ISC lineages are labelled with β-galactosidase (βgal, green) using Flp-mediated somatic recombination of a split lacZ gene (X-15-33/x-15-29), carrying or not *mRFP-Htt*^{Q138} (RU486-inducible ISC/EB driver 5961::GS). ISC-derived clones express βgal (white) and are outlined by yellow lines. Yellow arrowheads point to single βgal-labeled ISCs. mRFP-Htt^{Q138} aggregates are shown in red (first panel) or white (second panel). anti-Armadillo antibody was used to stained cell membranes (green). **c**, Number of β-galactosidase-positive clones (i.e. lineages) per area in the posterior midgut of flies were quantified at the indicated timepoints. This quantification relates to Fig. 1d and Fig. S1b (**b**). . Means and s.e.m. of numbers of clones / gut area are shown (n = 11, 13, 9, 9, respectively). 1-way ANOVA with Sidak's multiple comparisons test: ns, not significant * P = 0.0301. **d**, Representative images of 3rd instar larvae wing imaginal discs carrying GFP-marked lineages (green) derived from ISCs expressing or not *mRFP-Htt*^{Q138} (red) (using a Flp-out strategy). **a, b, c**, Hoechst was used to stain DNA (blue), scale bars indicated in figure. **d**, Selective expression of *mRFP-Htt*^{Q138} in ISCs and EBs (*esg::Gal4*^{ts}) of 6-day-old flies for 24 h or infected for 8 h one week after *mRFP-Htt*^{Q138} expression (first graph) or infected for 8 h two weeks after *mRFP-Htt*^{Q138} expression (second graph). Flies were dissected at the specified time points and guts were subjected to immunohistochemistry with anti-phospho-Histone H3 (pH3) antibody to label mitotic ISCs. Means and s.e.m. of numbers of pH3+ cells per gut are shown (n=17, 17, 16, 19 (1 week-time point); and 117, 19, 6, 8 (2 weeks-time point), respectively). **e**, Experiment was done as in (**d**), with the exception that *mRFP-Htt*^{Q138} was expressed in EBs (*Su(H)::Gal4*^{ts}). Means and s.e.m. of numbers of pH3+ cells per gut are shown (n=18, 20, 16, 18 (1 week-time point); and 8, 8, 15, 15 (2 weeks-time point), respectively). **d**, **e**, 2-way ANOVA with Tukey's multiple comparisons test: ns, not significant, **** $P < 0.0001$, * $P < 0.01$. Genotypes in Table S1.

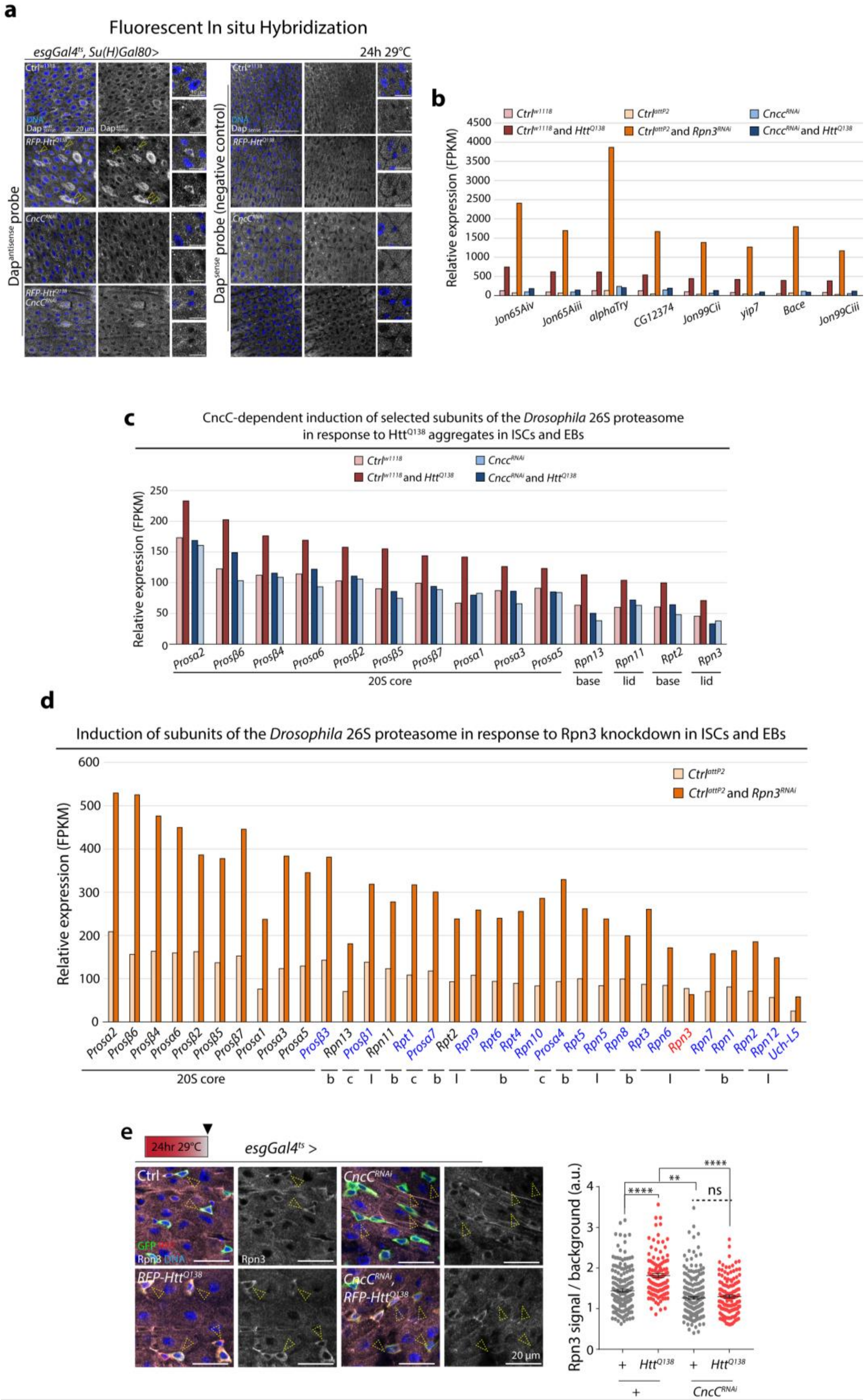

a
Fluorescent In situ Hybridization
esgGal4ts, Su(H)Gal80>
24h 29°C
Dapantisense probe
Dapsense probe (negative control)
Ctrlw1118
RFP-HttQ138
CncCRNAi
RFP-HttQ138 CncCRNAi
b
Relative expression (FPKM)
Ctrlw1118
Ctrlw1118 and HttQ138
CtrlattP2
CtrlattP2 and Rpn3RNAi
CnccRNAi
CnccRNAi and HttQ138
Jon65Aiv
Jon65Aiii
alphaTry
CG12374
Jon99Cii
yip7
Bace
Jon99Ciii
c
CncC-dependent induction of selected subunits of the Drosophila 26S proteasome in response to HttQ138 aggregates in ISCs and EBs
Relative expression (FPKM)
20S core
base
lid
d
Induction of subunits of the Drosophila 26S proteasome in response to Rpn3 knockdown in ISCs and EBs
Relative expression (FPKM)
CtrlattP2
CtrlattP2 and Rpn3RNAi
20S core
e
24hr 29°C
esgGal4ts >
Ctrl
CncCRNAi
RFP-HttQ138
CncCRNAi, RFP-HttQ138
Rpn3
20 µm
Rpn3 signal / background (a.u.)
****
**
ns
HttQ138
CncCRNAi

Supplementary Figure 2. a, Lower magnification of images shown in Fig. 4d. Fluorescent *In situ* hybridization of whole guts of 7-day-old flies after they were placed for 24 hours at 29°C to express or not in their ISCs RFP-Htt^{Q138} with or without $CncC^{RNAi}$. Shown in white is *dacapo* anti-sense probe labeled with 647(Cy5) fluorophore. *dacapo* sense probes was used as a negative control since it should not hybridize to *dacapo* mRNAs. DNA was stained with Hoechst (blue) and scale bars indicated in figure. **b**, Plotted is the relative expression as FPKM (Fragments Per Kilobase Million) for 8 proteases that are highly induced in sorted ISCs and EBs (esg+ cells) upon: *mRFP-*Htt^{Q138} expression for 2 days at 29°C (red bars) compared to its control ($Ctrl^{w1118}$, pink bars), and $Rpn3^{RNAi}$ expression for 4 days at 29°C (orange bars) compared to its control ($Ctrl^{attp2}$, yellow bars). The *RFP-*Htt^{Q138}-dependent induction is abolished when $CncC^{RNAi}$ is co-expressed in sorted esg+ cells: $CncC^{RNAi}$; *mRFP-*Htt^{Q138} expression for 2 days at 29°C (blue bars) compared to its control ($CncC^{RNAi}$, light blue bars). **c**, Plotted is the relative expression (FPKM values) for 14 genes encoding 20S core, base, or lid components of the *Drosophila* 26S proteasome that are highly induced in sorted *esg*+ (i.e. ISCs and EBs) cells upon *mRFP-*Htt^{Q138} expression for 2 days at 29°C (red bars) compared to its control ($Ctrl^{w1118}$, pink bars). The *mRFP-*Htt^{Q138}-dependent induction is abolished when $CncC^{RNAi}$ is co-expressed in sorted *esg*+ cells: $CncC^{RNAi}$; *mRFP-*Htt^{Q138} expression for 2 days at 29°C (blue bars) compared to its control ($CncC^{RNAi}$, light blue bars). **d**, Plotted is the relative expression (FPKM values) for 33 genes encoding 20S core, base (b), or lid (l) components of the *Drosophila* 26S proteasome that are highly induced in sorted *esg*+ cells upon $Rpn3^{RNAi}$ expression for 4 d at 29°C (orange bars) compared to its control ($Ctrl^{attp2}$, yellow bars). **e**, Representative area in the posterior midgut of flies expressing or not *mRFP-*Htt^{Q138} aggregates (red) in ISCs and EBs (green, *esg::*$Gal4^{ts}$). Guts were subjected to immunohistochemistry with anti-Rpn3 antibody (white) and stained with Hoechst (blue, DNA). Scale bars indicated in figure. Means and s.e.m. of Rpn3 (Cy5) signal normalized to background in GFP+ cells (n=181, 108, 214, and 145). 1-way ANOVA with Sidak's multiple comparisons test: ns, not significant, **** $P < 0.0001$, ** $P = 0.0014$.

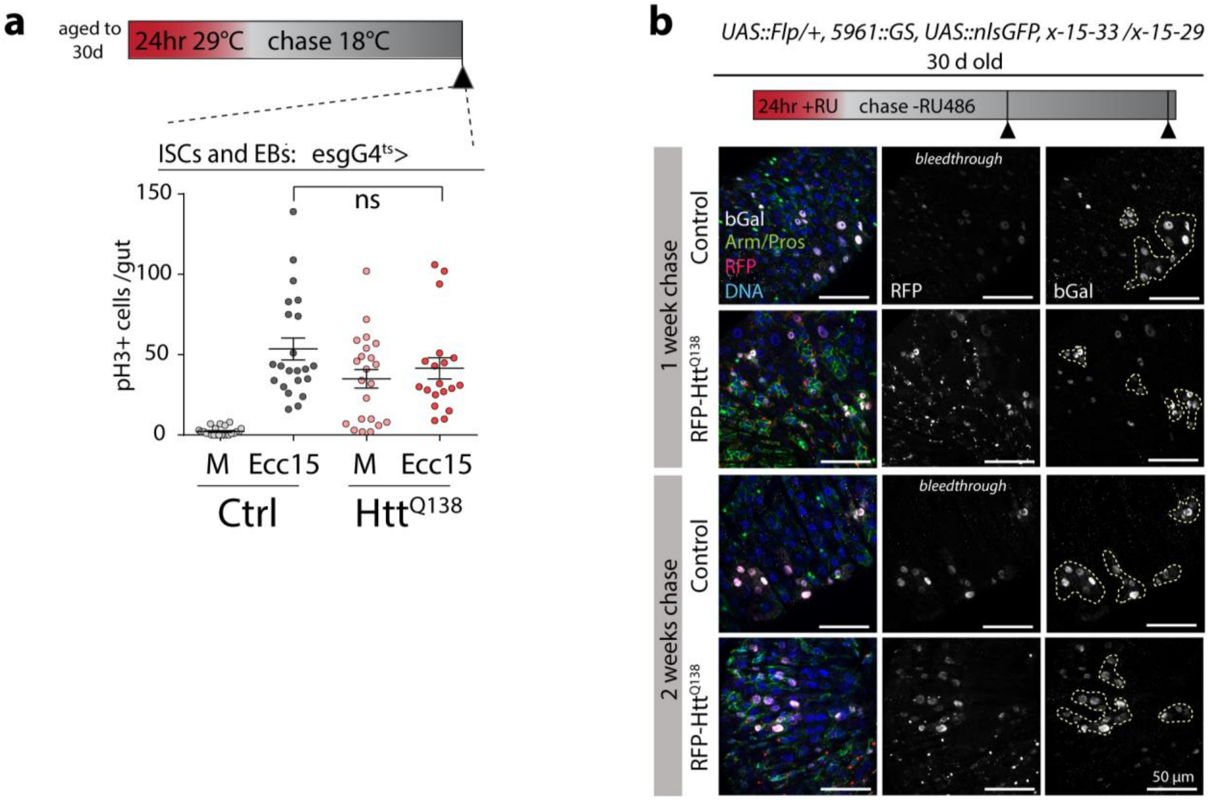

Supplementary Figure 3. a, Selective expression for 24 h at 29°C of *mRFP-Htt*^{Q138} in ISCs and EBs (*esg::Gal4*^{ts}) from 30-day-old flies followed by a 1-week chase period at 18°C and later an 8 h *Ecc15* infection. Intestines were immunostained with anti-pH3 antibody to label mitotic ISCs. Means and s.e.m. of the number of pH3+ cells per gut (n = 23, 22, 22, 19 guts, respectively). 2-way ANOVA with Tukey's multiple comparisons test: ns, not significant. **b**, Lower magnification of Flp-out lineages presented in Fig. 4C. ISC-derived clones express βgal (white) and are outlined by yellow lines. mRFP-Htt^{Q138} aggregates are shown in red (first panel) or white (second panel). anti-Armadillo antibody was used to stained cell membranes and anti-prospero antibody was used to detect enteroendocrine cells (both markers are shown in green in the first panel). Hoechst (blue) was used to label DNA. Scale bar indicated in figure.

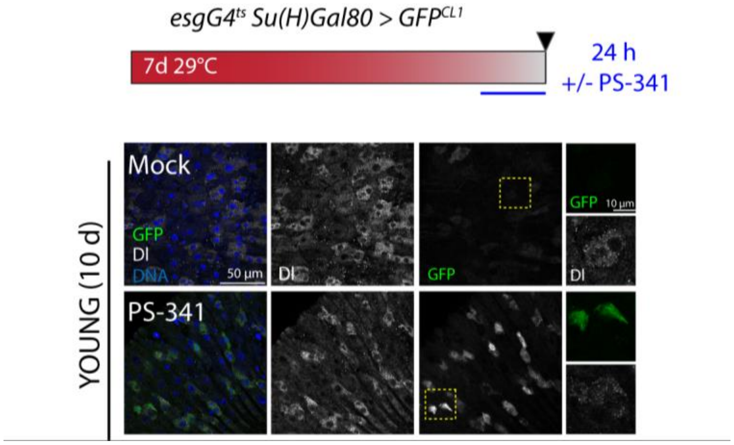

Supplementary Figure 4. Representative images of the posterior midgut of young flies (10-days old) after immunostaining using anti-Dl (gray: 1st and 2nd panel and lower inset). Hoechst (blue) was used to label DNA. As a positive control for the GFP^{CL1} proteasome activity reporter, flies (*esg::Gal4^{ts}, Su(H)::Gal80)* carrying a transgene expressing GFP^{CL1} (green: 1st panel and top inset: gray: 3rd panel) were placed for 6 days at 29°C and then treated for 24 h with food supplemented or not with 40 µM PS-341 (proteasome inhibitor). The GFP signal in Dl+ cells was quantified and is shown in Fig. 6a. Scale bar indicated in figure.

Supplementary Table 1. Genotypes of flies used in each figure panel.

**Fig. #**	**Genotype**
Fig. 1	
1a	w^{1118}/y^{1},sc*,v^{1}; esg::Gal4, UAS::GFP, tub::Gal80^{ts}/+; UAS-Flp, Actin>CD2>Gal4/attP2<br>where attP2 = P{CaryP}attP2<br><br>w^{1118}/y^{1},sc*,v^{1}; esg::Gal4, UAS::GFP, tub::Gal80^{ts}/+ ; UAS-Flp, Actin>CD2>Gal4/UAS::Rpn3^{RNAi}<br>where Rpn3^{RNAi} = P{TRiP.HM05247}attP2
1b	w^{1118}/y^{1},sc*,v^{1}; esg::Gal4, UAS::GFP, tub::Gal80^{ts}/+; UAS-Flp, Actin>CD2>Gal4/attP2<br>where attP2 = P{CaryP}attP2<br><br>w^{1118}/y^{1},sc*,v^{1}; esg::Gal4, UAS::GFP, tub::Gal80^{ts}/+ ; UAS-Flp, Actin>CD2>Gal4/UAS::Rpn3^{RNAi}<br>where Rpn3^{RNAi} = P{TRiP.HM05247}attP2<br><br>w^{1118}/y^{1},sc*,v^{1}; esg::Gal4, UAS::GFP, tub::Gal80^{ts}/+ ; UAS-Flp, Actin>CD2>Gal4/UAS::Rpn2^{RNAi}<br>where Rpn2^{RNAi} = P{TRiP.HMS00533}attP2<br><br>w^{1118}/y^{1},sc*,v^{1}; esg::Gal4, UAS::GFP, tub::Gal80^{ts}/+ ; UAS-Flp, Actin>CD2>Gal4/UAS::Rpt6R^{RNAi}<br>where RPT6R^{RNAi} = P{TRiP.HMS01330}attP2
1c	w^{1118}; esg::Gal4, UAS::2xYFP/+; Su(H)GBE::Gal80, tub::Gal80^{ts}/UAS::mRFP-Htt^{Q138}
1d	w^{1118}; UAS::Flp/5961::GS, UAS::nlsGFP, x-15-33 /x-15-29 ; +/+<br><br>w^{1118}; UAS::Flp/5961::GS, UAS::nlsGFP, x-15-33 /x-15-29; UAS::mRFP-Htt^{Q138}/+
1e -1g	w^{1118}; esg::Gal4, UAS::2xYFP/+; Su(H)GBE::Gal80, tub::Gal80^{ts}/+<br><br>w^{1118}; esg::Gal4, UAS::2xYFP/+; Su(H)GBE::Gal80, tub::Gal80^{ts}/UAS::mRFP-Htt^{Q138}
Fig. 2	
2a	w^{1118}; esg::Gal4, UAS::2xYFP/+; Su(H)GBE::Gal80, tub::Gal80^{ts}/+<br><br>w^{1118}; esg::Gal4, UAS::2xYFP/+; Su(H)GBE::Gal80, tub::Gal80^{ts}/UAS::mRFP-Htt^{Q138}<br><br>w^{1118}; esg::Gal4, UAS::2xYFP/UAS::CncC^{RNAi}; Su(H)GBE::Gal80, tub::Gal80^{ts}/+<br><br>w^{1118}; esg::Gal4, UAS::2xYFP/UAS::CncC^{RNAi}; Su(H)GBE::Gal80, tub::Gal80^{ts}/UAS::mRFP-Htt^{Q138}<br>where CncC^{RNAi} = P{KK101639}VIE-260B (from VDRC# 108127)<br><br>w^{1118}; esg::Gal4, UAS::2xYFP/UAS::Keap1HA; Su(H)GBE::Gal80, tub::Gal80^{ts}/+<br><br>w^{1118}; esg::Gal4, UAS::2xYFP/UAS::Keap1HA; Su(H)GBE::Gal80, tub::Gal80^{ts}/UAS::mRFP-Htt^{Q138}
2b	w^{1118}; esg::Gal4, UAS::2xYFP/+; Su(H)GBE::Gal80, tub::Gal80^{ts}/+<br><br>w^{1118}; esg::Gal4, UAS::2xYFP/+; Su(H)GBE::Gal80, tub::Gal80^{ts}/UAS::mRFP-Htt^{Q138}

	w^{1118}; esg::Gal4, UAS::2xYFP/UAS::CncC^{RNAi}; Su(H)GBE::Gal80, tub::Gal80^{ts}/+<br><br>w^{1118}; esg::Gal4, UAS::2xYFP/UAS::CncC^{RNAi}; Su(H)GBE::Gal80, tub::Gal80^{ts}/UAS::mRFP-Htt^{Q138}<br>where CncC^{RNAi} = P{KK101639}VIE-260B (from VDRC# 108127)
2c	w^{1118}; esg::Gal4, UAS::GFP, tub::Gal80^{ts}/+; UAS-Flp, Actin>CD2>Gal4/ UAS::mRFP-Htt^{Q138}<br><br>w^{1118}; esg::Gal4, UAS::GFP, tub::Gal80^{ts}/+; UAS-Flp, Actin>CD2>Gal4/+<br><br>w^{1118}; esg::Gal4, UAS::GFP, tub::Gal80^{ts}/ UAS::Keap1HA; UAS-Flp, Actin>CD2>Gal4/+<br><br>w^{1118}; esg::Gal4, UAS::GFP, tub::Gal80^{ts}/ UAS::Keap1HA; UAS-Flp, Actin>CD2>Gal4/ UAS::mRFP-Htt^{Q138}<br><br>w^{1118}/ y^{1},sc*,v^{1}; esg::Gal4, UAS::GFP, tub::Gal80^{ts}/UAS::CncC^{RNAi}; UAS-Flp, Actin>CD2>Gal4/+<br><br>w^{1118}/ y^{1},sc*,v^{1}; esg::Gal4, UAS::GFP, tub::Gal80^{ts}/UAS::CncC^{RNAi}; UAS-Flp, Actin>CD2>Gal4/ UAS::mRFP-Htt^{Q138}<br>where CncC^{RNAi} = P{KK101639}VIE-260B (from VDRC# 108127)
2d	Immunohistochemistry:<br><br>w^{1118}/y,w^{1118}; esg::Gal4, UAS::GFP, tub::Gal80^{ts}/attP; UAS::mRFP-Htt^{Q138}/+<br>where attP = P{empty}VIE-260B landing site (from VDRC# 60100)<br><br>w^{1118}/y,w^{1118}; esg::Gal4, UAS::GFP, tub::Gal80^{ts}/ CncC^{RNAi} ; UAS::mRFP-Htt^{Q138}/+<br>where CncC^{RNAi} = P{KK101639}VIE-260B (from VDRC# 108127)<br><br>In graph:<br><br>w^{1118}/w^{1118}; esg::Gal4, UAS::GFP, tub::Gal80^{ts}/+; UAS::mRFP-Htt^{Q138}/+<br><br>w^{1118}/y,w^{1118}; esg::Gal4, UAS::GFP, tub::Gal80^{ts}/ CncC^{RNAi} ; UAS::mRFP-Htt^{Q138}/+<br>where CncC^{RNAi} = P{KK101639}VIE-260B (from VDRC# 108127)<br><br>w^{1118}/w^{1118}; esg::Gal4, UAS::GFP, tub::Gal80^{ts}/UAS::Keap1HA; UAS::mRFP-Htt^{Q138}/+<br><br>w^{1118}/w^{1118}; esg::Gal4, UAS::GFP, tub::Gal80^{ts}/UAS::Keap1HA; UAS::mRFP-Htt^{Q138}/cnc^{VL110}
2e	w^{1118}; esg::Gal4, UAS::2xYFP/GstD1::LacZ; Su(H)GBE::Gal80, tub::Gal80^{ts}/+<br><br>w^{1118}; esg::Gal4, UAS::2xYFP/ GstD1::LacZ; Su(H)GBE::Gal80, tub::Gal80^{ts}/UAS::mRFP-Htt^{Q138}
2f	w^{1118}; esg::Gal4, UAS::2xYFP/GstD1::LacZ; Su(H)GBE::Gal80, tub::Gal80^{ts}/ UAS::Rpn3^{RNAi}<br>where Rpn3^{RNAi} = P{TRiP.HM05247}attP2
Fig. 3	
3a	For 2 replicates:<br><br>w^{1118}; esg::Gal4, UAS::2xYFP/+; Su(H)GBE::Gal80, tub::Gal80^{ts}/+

	w^{1118}; esg::Gal4, UAS::2xYFP/+; Su(H)GBE::Gal80, tub::Gal80^{ts}/UAS::mRFP-Htt^{Q138}<br><br>w^{1118}; esg::Gal4, UAS::2xYFP/UAS::Atg8a^{RNAi}; Su(H)GBE::Gal80, tub::Gal80^{ts}/+<br><br>w^{1118}; esg::Gal4, UAS::2xYFP/UAS::Atg8a^{RNAi}; Su(H)GBE::Gal80, tub::Gal80^{ts}/UAS::mRFP-Htt^{Q138}<br>where Atg8a^{RNAi} = P{KK102155}VIE-260B (from VDRC# 109654)<br><br>For one replicate:<br><br>w^{1118}; esg::Gal4, UAS::GFP/+; tub::Gal80^{ts}/+<br><br>w^{1118}; esg::Gal4, UAS::GFP/+; tub::Gal80^{ts}/UAS::mRFP-Htt^{Q138}<br><br>w^{1118}; esg::Gal4, UAS::GFP/UAS::Atg8a^{RNAi}; tub::Gal80^{ts}/+<br><br>w^{1118}; esg::Gal4, UAS::GFP/UAS::Atg8a^{RNAi}; tub::Gal80^{ts}/UAS::mRFP-Htt^{Q138}<br>where Atg8a^{RNAi} = P{KK102155}VIE-260B (from VDRC# 109654)
3b	w^{1118}; esg::Gal4, UAS::2xYFP/+; Su(H)GBE::Gal80, tub::Gal80^{ts}/+<br><br>w^{1118}; esg::Gal4, UAS::2xYFP/+; Su(H)GBE::Gal80, tub::Gal80^{ts}/UAS::mRFP-Htt^{Q138}<br><br>w^{1118}; esg::Gal4, UAS::2xYFP/dap^{4}; Su(H)GBE::Gal80, tub::Gal80^{ts}/+<br><br>w^{1118}; esg::Gal4, UAS::2xYFP/dap^{4}; Su(H)GBE::Gal80, tub::Gal80^{ts}/UAS::mRFP-Htt^{Q138}
3c	w^{1118}; esg::Gal4, UAS::GFP, tub::Gal80^{ts}/+ ; +/+<br><br>w^{1118}; esg::Gal4, UAS::GFP, tub::Gal80^{ts}/+ ; UAS::mRFP-Htt^{Q138}/+<br><br>w^{1118}; esg::Gal4, UAS::GFP, tub::Gal80^{ts}/+ ; UAS::dap^{RNAi}/+<br><br>w^{1118}; esg::Gal4, UAS::GFP,tub::Gal80^{ts}/+; UAS::mRFP-Htt^{Q138}/UAS::dap^{RNAi}<br>where dap^{RNAi} = P{TRiP.HMS01610}attP2 (from Bloomington 36720)
3d	w^{1118}/y,w^{1118}; esg::Gal4, UAS::GFP, tub::Gal80^{ts}/attP ; UAS::mRFP-Htt^{Q138}/+<br>where attP = P{empty}VIE-260B landing site (from VDRC# 60100)<br><br>w^{1118}/y,w^{1118}; esg::Gal4, UAS::GFP,tub::Gal80^{ts}/ Atg8a^{RNAi}; UAS::mRFP-Htt^{Q138}/+<br>where Atg8a^{RNAi} = P{KK102155}VIE-260B (from VDRC# 109654)
3e	w^{1118}; esg::Gal4, UAS::GFP, tub::Gal80^{ts}/+; UAS-Flp, Actin>CD2>Gal4/ UAS::mRFP-Htt^{Q138}<br><br>w^{1118}; esg::Gal4, UAS::GFP, tub::Gal80^{ts}/+ ; UAS-Flp, Actin>CD2>Gal4/+<br><br>w^{1118}; esg::Gal4, UAS::GFP, tub::Gal80^{ts}/UAS::Atg8a^{RNAi}; UAS-Flp, Actin>CD2>Gal4/ UAS::mRFP-Htt^{Q138}<br><br>w^{1118}; esg::Gal4, UAS::GFP, tub::5Gal80^{ts}/ UAS::Atg8a^{RNAi}; UAS-Flp, Actin>CD2>Gal4/+<br>where Atg8a^{RNAi} = P{KK102155}VIE-260B (from VDRC# 109654)
3f	w^{1118}; esg::Gal4, UAS::GFP, tub::Gal80^{ts}/+ ; UAS::mRFP-Htt^{Q138}/+

	w^{1118}; esg::Gal4, UAS::GFP,tub::Gal80^{ts}/ dap^{4}; UAS::mRFP-Htt^{Q138}/+<br><br>w^{1118}; esg::Gal4, UAS::GFP, tub::Gal80^{ts}/+ ; UAS::mRFP-Htt^{Q138}/+<br><br>w^{1118}; esg::Gal4, UAS::GFP,tub::Gal80^{ts}/+; UAS::mRFP-Htt^{Q138}/UAS::dap^{RNAi}<br>where dap^{RNAi} = P{TRiP.HMS01610}attP2 (from Bloomington 36720)
3h	w^{1118}; esg::Gal4, UAS::GFP, tub::Gal80^{ts}/+ ; +/+<br><br>w^{1118}; esg::Gal4, UAS::GFP, tub::Gal80^{ts}/+ ; UAS::mRFP-Htt^{Q138}/+<br><br>w^{1118}; esg::Gal4, UAS::GFP, tub::Gal80^{ts}/+ ; UAS::dap^{RNAi}/+<br><br>w^{1118}; esg::Gal4, UAS::GFP,tub::Gal80^{ts}/+; UAS::mRFP-Htt^{Q138}/UAS::dap^{RNAi}<br>where dap^{RNAi} = P{TRiP.HMS01610}attP2 (from Bloomington 36720)
Fig. 4	
4a	w^{1118}/y^{1},sc*,v^{1}; esg::Gal4, UAS::2xYFP/+; Su(H)GBE::Gal80, tub::Gal80^{ts}/UAS::mCherry^{RNAi}<br><br>w^{1118}/y^{1},sc*,v^{1}; esg::Gal4, UAS::2xYFP/+; Su(H)GBE::Gal80, tub::Gal80^{ts}/UAS::Rpn3^{RNAi}<br>where Rpn3^{RNAi} = P{TRiP.HM05247}attP2
4b	w^{1118}; esg::Gal4, UAS::GFP, GstD1::LacZ/+; tub::Gal80^{ts}/UAS::mRFP-Htt^{Q138}<br><br>w^{1118}; esg::Gal4, UAS::GFP, GstD1::LacZ /UAS::Atg8a^{RNAi}; tub::Gal80^{ts}/UAS::mRFP-Htt^{Q138}<br>where Atg8a^{RNAi} = P{KK102155}VIE-260B (from VDRC# 109654)
4c	w^{1118}/y^{1},sc*,v^{1}; esg::Gal4, UAS::2xYFP/+; Su(H)GBE::Gal80, tub::Gal80^{ts}/attP2<br>where attP2 = P{CaryP}attP2<br><br>w^{1118}/y^{1},sc*,v^{1}; esg::Gal4, UAS::2xYFP/+; Su(H)GBE::Gal80, tub::Gal80^{ts}/UAS::Rpn3^{RNAi}<br>where Rpn3^{RNAi} = P{TRiP.HM05247}attP2<br><br>w^{1118}; esg::Gal4, UAS::2xYFP/+; Su(H)GBE::Gal80, tub::Gal80^{ts}/+<br><br>w^{1118}; esg::Gal4, UAS::2xYFP/+; Su(H)GBE::Gal80, tub::Gal80^{ts}/UAS::mRFP-Htt^{Q138}<br><br>w^{1118}; esg::Gal4, UAS::GFP/UAS:: CncC^{RNAi}; tub::Gal80^{ts}/+<br><br>w^{1118}; esg::Gal4, UAS::GFP/ UAS:: CncC^{RNAi}; tub::Gal80^{ts}/UAS::mRFP-Htt^{Q138}<br>where CncC^{RNAi} = P{KK101639}VIE-260B (from VDRC# 108127)
4d	w^{1118}; esg::Gal4, UAS::2xYFP/+; Su(H)GBE::Gal80, tub::Gal80^{ts}/+<br><br>w^{1118}; esg::Gal4, UAS::2xYFP/+; Su(H)GBE::Gal80, tub::Gal80^{ts}/UAS::mRFP-Htt^{Q138}<br><br>w^{1118}; esg::Gal4, UAS::GFP/UAS:: CncC^{RNAi}; tub::Gal80^{ts}/+<br><br>w^{1118}; esg::Gal4, UAS::GFP/ UAS:: CncC^{RNAi}; tub::Gal80^{ts}/UAS::mRFP-Htt^{Q138}<br>where CncC^{RNAi} = P{KK101639}VIE-260B (from VDRC# 108127)

4e	w^{1118}; esg::Gal4, UAS::nlsGFP/+, tub::Gal80^{ts}/+<br><br>w^{1118}; esg::Gal4, UAS::nlsGFP, tub::Gal80^{ts}/+; UAS::mRFP-Htt^{Q138}/+<br><br>w^{1118}; esg::Gal4, UAS::nlsGFP/+, tub::Gal80^{ts}/ Atg8a^{EP362}; +/+<br><br>w^{1118}; esg::Gal4, UAS::nlsGFP/+, tub::Gal80^{ts}/ Atg8a^{EP362}; UAS::mRFP-Htt^{Q138}/+<br><br>w^{1118}; esg::Gal4, UAS::nlsGFP/+, tub::Gal80^{ts}/+ ; UAS::Atg1/+<br><br>w^{1118}; esg::Gal4, UAS::nlsGFP/+, tub::Gal80^{ts}/+ ; UAS::Atg1/ UAS::mRFP-Htt^{Q138}/+
4f	w^{1118}; esg::Gal4, UAS::GFP/+; tub::Gal80^{ts}/+<br><br>w^{1118}; esg::Gal4, UAS::GFP/+; tub::Gal80^{ts}/UAS::mRFP-Htt^{Q138}<br><br>w^{1118}/y^{1},sc*,v^{1}; esg::Gal4, UAS::GFP/+; tub::Gal80^{ts}/attP2<br>where attP2 = P{CaryP}attP2<br><br>w^{1118}/y^{1},sc*,v^{1}; esg::Gal4, UAS::GFP/+; tub::Gal80^{ts}/UAS::Rpn3^{RNAi}<br>where Rpn3^{RNAi} = P{TRiP.HM05247}attP2
4g	w^{1118}; esg::Gal4, UAS::GFP/+; tub::Gal80^{ts}/+<br><br>w^{1118}; esg::Gal4, UAS::GFP/+; tub::Gal80^{ts}/UAS::mRFP-Htt^{Q138}<br><br>w^{1118}/y,w^{1118}; esg::Gal4, UAS::GFP/UAS:: CncC^{RNAi}; tub::Gal80^{ts}/+<br><br>w^{1118}/y,w^{1118}; esg::Gal4, UAS::GFP/ UAS:: CncC^{RNAi}; tub::Gal80^{ts}/UAS::mRFP-Htt^{Q138}<br>where CncC^{RNAi} = P{KK101639}VIE-260B (from VDRC# 108127)
Fig. 5	
5a	w^{1118}/mChFP::Rho1; esg::Gal4, UAS::2xYFP/+; Su(H)GBE::Gal80/+
5b	w^{1118}; esg::Gal4/UAS::GFP^{CL1}; Su(H)GBE::Gal80, tub::Gal80^{ts}/+
5c	w^{1118}; esg::Gal4, UAS::2xYFP/+; Su(H)GBE::Gal80, tub::Gal80^{ts}/UAS::mRFP-Htt^{Q138}
5d	w^{1118}; esg::Gal4, UAS::2xYFP/+; Su(H)GBE::Gal80, tub::Gal80^{ts}/+<br><br>w^{1118}; esg::Gal4, UAS::2xYFP/+; Su(H)GBE::Gal80, tub::Gal80^{ts}/UAS::mRFP-Htt^{Q138}<br><br>w^{1118}; Su(H)GBE::Gal4, UAS::CD8GFP/+; tub::Gal80^{ts}/+<br><br>w^{1118}; Su(H)GBE::Gal4, UAS::CD8GFP/+; tub::Gal80^{ts}/ UAS::mRFP-Htt^{Q138}
5e	w^{1118}; UAS::Flp/5961::GS, UAS::nlsGFP, x-15-33 /x-15-29 ; +/+<br><br>w^{1118}; UAS::Flp/5961::GS, UAS::nlsGFP, x-15-33 /x-15-29; UAS::mRFP-Htt^{Q138}/+
5f	w^{1118}; esg::Gal4, UAS::2xYFP/+; Su(H)GBE::Gal80, tub::Gal80^{ts}/+<br><br>w^{1118}; esg::Gal4, UAS::2xYFP/+; Su(H)GBE::Gal80, tub::Gal80^{ts}/UAS::mRFP-Htt^{Q138}
Fig. 6	

6a	w^{1118}; esg::Gal4/UAS::GFP^{CL1}; Su(H)GBE::Gal80, tub::Gal80^{ts}/+
6b	w^{1118}/mChFP::Rho1; esg::Gal4, UAS::2xYFP/+; Su(H)GBE::Gal80/+
6c	w^{1118}; esg::Gal4, UAS::2xYFP/+; Su(H)GBE::Gal80, tub::Gal80^{ts}/+<br><br>w^{1118}; esg::Gal4, UAS::2xYFP/UAS::CncCFL2; Su(H)GBE::Gal80, tub::Gal80^{ts}/+<br><br>w^{1118}; esg::Gal4, UAS::2xYFP/Atg8a^{EP362}; Su(H)GBE::Gal80, tub::Gal80^{ts}/+
6d	w^{1118}; esg::Gal4, UAS::2xYFP/+; Su(H)GBE::Gal80, tub::Gal80^{ts}/UAS::mRFP-Htt^{Q138}
6e	w^{1118}; esg::Gal4, UAS::2xYFP/UAS::luciferase; Su(H)GBE::Gal80, tub::Gal80^{ts}/+<br><br>w^{1118}; esg::Gal4, UAS::2xYFP/+; Su(H)GBE::Gal80, tub::Gal80^{ts}/UAS::mRFP-Htt^{Q138}
6f	w^{1118}; esg::Gal4, UAS::2xYFP/+; Su(H)GBE::Gal80, tub::Gal80^{ts}/+<br><br>w^{1118}; esg::Gal4, UAS::2xYFP/UAS::CncCFL2; Su(H)GBE::Gal80, tub::Gal80^{ts}/+<br><br>w^{1118}; esg::Gal4, UAS::2xYFP/UAS::Keap1HA; Su(H)GBE::Gal80, tub::Gal80^{ts}/+<br><br>w^{1118}; esg::Gal4, UAS::2xYFP/Atg8a^{EP362}; Su(H)GBE::Gal80, tub::Gal80^{ts}/+
6g, h	w^{1118}; esg::Gal4, UAS::2xYFP/UAS::luciferase; Su(H)GBE::Gal80, tub::Gal80^{ts}/+<br><br>w^{1118}; esg::Gal4, UAS::2xYFP/+; Su(H)GBE::Gal80, tub::Gal80^{ts}/UAS::mRFP-Htt^{Q138}
	SUPPLEMENTARY FIGURES
S1	
S1a	w^{1118}/y^{1},sc*,v^{1}; esg::Gal4, UAS::GFP, tub::Gal80^{ts}/+ ; UAS-Flp, Actin>CD2>Gal4/UAS::Rpn2^{RNAi}<br>where Rpn2^{RNAi} = P{TRiP.HMS00533}attP2<br><br>w^{1118}/y^{1},sc*,v^{1}; esg::Gal4, UAS::GFP, tub::Gal80^{ts}/+ ; UAS-Flp, Actin>CD2>Gal4/UAS::Rpt6R^{RNAi}<br>where Rpt6R^{RNAi} = P{TRiP.HMS01330}attP2
S1b, c	w^{1118}; UAS::Flp/5961::GS, UAS::nlsGFP, x-15-33 /x-15-29 ; +/+<br><br>w^{1118}; UAS::Flp/5961::GS, UAS::nlsGFP, x-15-33 /x-15-29; UAS::mRFP-Htt^{Q138}/+
S1d	w^{1118}; esg::Gal4, UAS::GFP, tub::Gal80^{ts}/+; UAS-Flp, Actin>CD2>Gal4/ +<br><br>w^{1118}; esg::Gal4, UAS::GFP, tub::Gal80^{ts}/+; UAS-Flp, Actin>CD2>Gal4/ UAS::mRFP-Htt^{Q138}
S1g	w^{1118}; esg::Gal4, UAS::nlsGFP, tub::Gal80^{ts}/+ ; +/+<br><br>w^{1118}; esg::Gal4, UAS::nlsGFP, tub::Gal80^{ts}/+; UAS::mRFP-Htt^{Q138}/+
S1f	w^{1118}; Su(H)GBE::Gal4, UAS::CD8GFP/+; tub::Gal80^{ts}/+<br><br>w^{1118}; Su(H)GBE::Gal4, UAS::CD8GFP/+; tub::Gal80^{ts}/ UAS::mRFP-Htt^{Q138}

S2	
S2a	w^{1118}; esg::Gal4, UAS::2xYFP/+; Su(H)GBE::Gal80, tub::Gal80^{ts}/+<br><br>w^{1118}; esg::Gal4, UAS::2xYFP/+; Su(H)GBE::Gal80, tub::Gal80^{ts}/UAS::mRFP-Htt^{Q138}<br><br>w^{1118}; esg::Gal4, UAS::GFP/UAS:: CncC^{RNAi}; tub::Gal80^{ts}/+<br><br>w^{1118}; esg::Gal4, UAS::GFP/ UAS:: CncC^{RNAi}; tub::Gal80^{ts}/UAS::mRFP-Htt^{Q138}<br>where CncC^{RNAi} = P{KK101639}VIE-260B (from VDRC# 108127)
S2b	w^{1118}; esg::Gal4, UAS::GFP/+; tub::Gal80^{ts}/+<br><br>w^{1118}; esg::Gal4, UAS::GFP/+; tub::Gal80^{ts}/UAS::mRFP-Htt^{Q138}<br><br>w^{1118}/y^{1},sc*,v^{1}; esg::Gal4, UAS::GFP/+; tub::Gal80^{ts}/attP2<br>where attP2 = P{CaryP}attP2<br><br>w^{1118}/y^{1},sc*,v^{1}; esg::Gal4, UAS::GFP/+; tub::Gal80^{ts}/UAS::Rpn3^{RNAi}<br>where Rpn3^{RNAi} = P{TRiP.HM05247}attP2<br><br>w^{1118}/y,w^{1118}; esg::Gal4, UAS::GFP/UAS:: CncC^{RNAi}; tub::Gal80^{ts}/+<br><br>w^{1118}/y,w^{1118}; esg::Gal4, UAS::GFP/ UAS:: CncC^{RNAi}; tub::Gal80^{ts}/UAS::mRFP-Htt^{Q138}<br>where CncC^{RNAi} = P{KK101639}VIE-260B (from VDRC# 108127)
S2c	w^{1118}; esg::Gal4, UAS::GFP/+; tub::Gal80^{ts}/+<br><br>w^{1118}; esg::Gal4, UAS::GFP/+; tub::Gal80^{ts}/UAS::mRFP-Htt^{Q138}<br><br>w^{1118}/y,w^{1118}; esg::Gal4, UAS::GFP/UAS:: CncC^{RNAi}; tub::Gal80^{ts}/+<br><br>w^{1118}/y,w^{1118}; esg::Gal4, UAS::GFP/ UAS:: CncC^{RNAi}; tub::Gal80^{ts}/UAS::mRFP-Htt^{Q138}<br>where CncC^{RNAi} = P{KK101639}VIE-260B (from VDRC# 108127)
S2d	w^{1118}/y^{1},sc*,v^{1}; esg::Gal4, UAS::GFP/+; tub::Gal80^{ts}/attP2<br>where attP2 = P{CaryP}attP2<br><br>w^{1118}/y^{1},sc*,v^{1}; esg::Gal4, UAS::GFP/+; tub::Gal80^{ts}/UAS::Rpn3^{RNAi}<br>where Rpn3^{RNAi} = P{TRiP.HM05247}attP2
S2e	w^{1118}/y^{1},sc*,v^{1}; esg::Gal4, UAS::GFP/+; tub::Gal80^{ts}/attP2<br>where attP2 = P{CaryP}attP2<br><br>w^{1118}/y^{1},sc*,v^{1}; esg::Gal4, UAS::GFP/+; tub::Gal80^{ts}/UAS::Rpn3^{RNAi}<br>where Rpn3^{RNAi} = P{TRiP.HM05247}attP2
S3	
S3a	w^{1118}; esg::Gal4, UAS::nlsGFP, tub::Gal80^{ts}/+ ; +/+<br><br>w^{1118}; esg::Gal4, UAS::nlsGFP, tub::Gal80^{ts}/+; UAS::mRFP-Htt^{Q138}/+
S3b	w^{1118}; UAS::Flp/5961::GS, UAS::nlsGFP, x-15-33 /x-15-29 ; +/+<br><br>w^{1118}; UAS::Flp/5961::GS, UAS::nlsGFP, x-15-33 /x-15-29; UAS::mRFP-Htt^{Q138}/+

S4	
	w^{1118}; esg::Gal4/UAS::GFP^{CL1}; Su(H)GBE::Gal80, tub::$Gal80^{ts}$/+

Supplementary Methods

Larval imaginal discs dissection and staining. Parental crosses were set up and eggs hatched at 18°C to avoid transgene expression. Early second instar larvae (5 days after egg laying at 18°C) were heat shocked for 45 min at 37°C, and placed back at 18°C. Three days later 3rd instar larvae were dissected in PBS. Wing discs were fixed for 30 minutes at room temperature in a solution of 3.7% formaldehyde in PBS, and blocked in a solution containing 0.1% TritonX-100 and 10% Normal Goat Serum in PBS. Discs were quickly washed 3x at room temperature before washing overnight at 4°C with washing solution (0.1% TritonX-100 in PBS). Then discs were incubated for 15 minutes in a solution containing 1:1000 Hoechst in PBS (to stain DNA) and mounted in Vectashield (Vector laboratories Inc, H-1000).
