# Supplementary material for: Loss of a proteostatic checkpoint in intestinal stem cells contributes to age-related epithelial dysfunction
Source: Nat Commun. 2019 Mar 5;10:1050. doi: 10.1038/s41467-019-08982-9 (PMC6401111; doi:10.1038/s41467-019-08982-9)
Supplement: Supplementary file 3 — Reporting Summary [file 41467_2019_8982_MOESM3_ESM.pdf]

nature research

Corresponding author(s): *Double-blind peer review submissions: write DBPR and your manuscript number here instead of author names.*

Last updated by author(s): *YYYY-MM-DD*

Reporting Summary

Nature Research wishes to improve the reproducibility of the work that we publish. This form provides structure for consistency and transparency in reporting. For further information on Nature Research policies, see Authors & Referees and the Editorial Policy Checklist.

Statistics

For all statistical analyses, confirm that the following items are present in the figure legend, table legend, main text, or Methods section.

n/a	Confirmed	
☐	☒	The exact sample size (*n*) for each experimental group/condition, given as a discrete number and unit of measurement
☐	☒	A statement on whether measurements were taken from distinct samples or whether the same sample was measured repeatedly
☐	☒	The statistical test(s) used AND whether they are one- or two-sided<br>*Only common tests should be described solely by name; describe more complex techniques in the Methods section.*
☒	☐	A description of all covariates tested
☐	☒	A description of any assumptions or corrections, such as tests of normality and adjustment for multiple comparisons
☐	☒	A full description of the statistical parameters including central tendency (e.g. means) or other basic estimates (e.g. regression coefficient) AND variation (e.g. standard deviation) or associated estimates of uncertainty (e.g. confidence intervals)
☐	☒	For null hypothesis testing, the test statistic (e.g. *F*, *t*, *r*) with confidence intervals, effect sizes, degrees of freedom and *P* value noted<br>*Give P values as exact values whenever suitable.*
☒	☐	For Bayesian analysis, information on the choice of priors and Markov chain Monte Carlo settings
☒	☐	For hierarchical and complex designs, identification of the appropriate level for tests and full reporting of outcomes
☒	☐	Estimates of effect sizes (e.g. Cohen's *d*, Pearson's *r*), indicating how they were calculated

Our web collection on statistics for biologists contains articles on many of the points above.

Software and code

Policy information about availability of computer code

Data collection	no software was used
Data analysis	Data was analyze in GraphPad Prism v7.02

For manuscripts utilizing custom algorithms or software that are central to the research but not yet described in published literature, software must be made available to editors/reviewers. We strongly encourage code deposition in a community repository (e.g. GitHub). See the Nature Research guidelines for submitting code & software for further information.

Data

Policy information about availability of data

All manuscripts must include a data availability statement. This statement should provide the following information, where applicable:
- Accession codes, unique identifiers, or web links for publicly available datasets
- A list of figures that have associated raw data
- A description of any restrictions on data availability

Data availability. RNAseq data is available at Gene Expression Omnibus (GEO) database under the accession number GSE125385 [https://www.ncbi.nlm.nih.gov/geo/query/acc.cgi?acc=GSE125385].

Field-specific reporting

Please select the one below that is the best fit for your research. If you are not sure, read the appropriate sections before making your selection.

☒ Life sciences ☐ Behavioural & social sciences ☐ Ecological, evolutionary & environmental sciences

For a reference copy of the document with all sections, see nature.com/documents/nr-reporting-summary-flat.pdf

Life sciences study design

All studies must disclose on these points even when the disclosure is negative.

Sample size	No sample size calculation was done. Numbers used were based on previous published experimental paradigms.
Data exclusions	For the data generated after Ecc15 infection experiments we performed a Grubbs's Outlier test (also known as Extreme Studentized Seviate method) for each group before analyzing the data using a statistical test.
Replication	Key findings in the paper (i.e. aggregate clearance, Paraquat-induced mortality, lineage tracing, Oltipraz treatment) were repeated at least 2 times.
Randomization	Assignment was random. All experiments were done using a pool of siblings hatched at same times and raised under same conditions. At the indicated age flies were split randomly into the required groups for interventions.
Blinding	No blinding was done.

Reporting for specific materials, systems and methods

We require information from authors about some types of materials, experimental systems and methods used in many studies. Here, indicate whether each material, system or method listed is relevant to your study. If you are not sure if a list item applies to your research, read the appropriate section before selecting a response.

Materials & experimental systems

n/a	Involved in the study	
☐	☒	Antibodies
☒	☐	Eukaryotic cell lines
☒	☐	Palaeontology
☐	☒	Animals and other organisms
☒	☐	Human research participants
☒	☐	Clinical data

Methods

n/a	Involved in the study	
☒	☐	ChIP-seq
☒	☐	Flow cytometry
☒	☐	MRI-based neuroimaging

Antibodies

Antibodies used	Antibodies. Commercial primary antibodies and dilutions were used as following: mouse anti-mono- and polyubiquitinylated proteins, 1:100 (FK2 clone, Enzo Cat. No. BML-PW8810); rabbit antiphospho-Histone H3 Ser10, 1:1000 (EMD Millipore Cat. No. 06-570); mouse anti-Rpn3/PSMD3 (G-1), 1:500 (Santa Cruz Biotecnology Inc Cat. No. sc-393588); Rabbit anti-β-galactosidase (Cappel MP Biomedicals, 1:500); mouse anti-Armadillo (DSHB, 1:100), mouse anti-Prospero (DSHB, 1:100) and mouse anti-Dacapo (DSHB, 1:4). Rat anti-Delta antibody, 1:500, was a gift from M. Rand (University of Rochester, USA). Rabbit anti-Atg8a, 1:200, was a gift from G. Juhasz (Eotvos Lorand University, Hungary). Fluorescent secondary antibodies were from Jackson Immunoresearch.
Validation	Validation was done previously by us and others since these are commonly used and published antibodies.

Animals and other organisms

Policy information about studies involving animals; ARRIVE guidelines recommended for reporting animal research

Laboratory animals	For all studies we used adult females Drosophila melanogaster. Age used is indicated in Figures or Figure Legends. The only time we used 3rd Instar Drosophila melanogaster larvae was in Supplementary Figure 1d. Full genotypes available on Supplementary Table 1.
Wild animals	N/A
Field-collected samples	N/A

Ethics oversight	N/A

Note that full information on the approval of the study protocol must also be provided in the manuscript.
